# Signal mining and analysis of adverse events in children using growth hormones: A retrospective real-world study based on FAERS

**DOI:** 10.1097/MD.0000000000049264

**Published:** 2026-06-12

**Authors:** Wan Xu, Jianan Bao, Yao Fei

**Affiliations:** a Department of Pharmacy, The Fourth Affiliated Hospital of Soochow University, Suzhou Dushu Lake Hospital, Medical Center of Soochow University, Jiangsu Suzhou, China.

**Keywords:** adverse events, children, FAERS, growth hormone, signal mining

## Abstract

This study aims to analyze the adverse events (AEs) related to the use of growth hormone (GH) drugs in children through mining the FDA adverse event reporting system (FAERS) database, and provide references for clinical medication safety. Data on children under 18 years of age from the FAERS database, covering the first quarter of 2004 to the first quarter of 2024, were extracted via the OpenVigil 2.1 tool. The reporting odds ratio was employed to analyze AE signals related to GHs. A total of 10,559 AE reports for children using GHs were obtained, revealing 361 risk signals across 20 organ systems. The top 5 system organ categories on the basis of the number of reports were general disorders and administration site conditions (33.4%), investigations (19.97%), musculoskeletal and connective tissue disorders (13.47%), nervous system disorders (13.06%), and metabolism and nutrition disorders (3.55%). Among the positive signals not mentioned in the product labeling, the top 5 were elevated blood urea nitrogen/creatinine ratio, increased bone density, decreased vitamin D, sleep apnea syndrome, and papilloedema. The findings align with known AEs (e.g., injection site reactions, headaches) but also reveal unreported risks, emphasizing gaps in current labeling. High signal intensity for musculoskeletal and neurological events underscores the need for targeted monitoring. While tumor-related reports (3.28%) were noted, existing databases suggest no causal link, highlighting the limitations of FAERS in establishing causality. Racial bias in reporting (predominantly from the US) and voluntary reporting biases may affect generalizability. The signal intensity associated with muscle, skeletal, and neurological abnormalities in children via GHs is relatively high. Continuous monitoring and enhanced medication oversight are necessary.

## 1. Introduction

With improvements in people’s living standards, parents are increasingly paying attention to children’s height. The causes of short stature in children are complex and include growth hormone deficiency (GHD), idiopathic short stature, skeletal development abnormalities, and certain genetic syndromes.^[1]^ Among these diseases, GHD is an endocrine metabolic disease caused by insufficient secretion of growth hormone (GH) from the anterior pituitary.^[2]^ In the 1950s, exogenous GH was first used to treat children with GHD. Subsequently, recombinant human growth hormone (rhGH), synthesized in *Escherichia coli* or mammalian cell cultures, was introduced in the late 1980s, and its role in promoting growth and improving final height has been well recognized.^[3]^ After years of research, Turner syndrome, idiopathic short stature, Noonan syndrome, Prader-Willi syndrome, small for gestational age, and severe burns have been approved as indications for rhGH. With the continuous deepening of research, it has also been found to have beneficial effects on chronic kidney disease.^[4,5]^ While rhGH is widely used in the clinic, adverse drug reactions are related to bone health, glucose and lipid metabolism, and the nervous system.^[6]^ Existing studies have explained the causes of some common adverse reactions. Some studies suggest that GH promotes skeletal growth via insulin-like growth factor-1 but can lead to adverse effects such as scoliosis and slipped capital femoral epiphysis due to rapid growth and increased bone metabolism, although the overall incidence is low.^[7,8]^ GH antagonizes insulin, inhibiting glucose utilization and causing transient increases in blood glucose and related laboratory indices. However, studies have shown that GH does not increase the incidence of diabetes in children, although it may lead to earlier onset in those predisposed to diabetes in adulthood.^[9,10]^ Some reports indicate that GH can cause mild sodium and water retention, leading to benign intracranial hypertension, characterized by headaches, vomiting, and vision loss, which typically resolves quickly after discontinuation. This reaction may occur because growth hormone rapidly corrects cerebrospinal fluid shifts caused by long-term GH deficiency.^[11,12]^ Although there are many articles on the safety of growth hormone, there are still few safety evaluations based on real-world, comprehensive consideration and systematic analysis focusing on children’s application scenarios.

The FDA adverse event reporting system (FAERS) aims to ensure drug and medical device safety. It collects and analyzes adverse event (AE) data from healthcare professionals, patients, and manufacturers. Data include patient information and event details. FAERS data is publicly accessible through the FAERS public dashboard, allowing anyone to query and analyze the information.^[13]^ OpenVigil 2.1 is a software package for pharmacovigilance data analysis that can extract data from FAERS, perform various analyses including disproportionality analysis and safety comparison of drugs, and offer customizable options. This open-source program was developed by Christian-Albrechts-Universität zu Kiel, Germany.^[14]^ This study employs the FAERS database for data mining, analyzing, and summarizing pharmacovigilance information of all growth hormone-related drugs in children. By utilizing real-world evidence, our research aims to enhance the understanding of adverse reactions associated with growth hormones and provide information for clinical practice to promote safe and rational drug use.

## 2. Materials and methods

### 2.1. Data sources

The data for this study were obtained from the FAERS database, covering data from the first quarter of 2004 to the first quarter of 2024. Each quarterly dataset includes 7 subfiles containing basic case information, drug information, indications for use, adverse event information, clinical outcomes, reporting sources, and treatment duration.

### 2.2. Study design

This was a retrospective pharmacovigilance signal mining study based on the FAERS database. Time to AEs or serious outcomes was not calculated in this study, because the FAERS database lacks complete and standardized time information for the onset of AEs.

### 2.3. Data extraction and organization

This study utilized the drug vigilance tool OpenVigil 2.1, which was developed and validated by the University of Kiel in Germany, to query and extract data from FAERS. The generic names “human growth hormone” and “somatotropin” were used as search terms, with filtering criteria set for patients aged 0 to 18 years. The data were extracted and downloaded included patients’ ages, sex, medication information, outcomes, indications, reporters, and reporting cities. Growth hormone was treated as the primary suspected drug for data filtering and cleaning, with duplicate reports and reports related to other indications excluded. Finally, AE signals were organized and categorized via preferred terms and system organ-class from the 26.1 version of the medical dictionary for regulatory activities. This study used de-identified, publicly available data from the FAERS database. Therefore, institutional review board approval and informed consent were waived (see Ethics Statement).

### 2.4. Adverse event signal mining

The signal mining method employed in this study was the reporting odds ratio (ROR) from the disproportionality analysis. A 2 × 2 contingency table was used for calculations, where “*a*” represents the number of AE reports for the target drug, “*b*” represents the number of reports for other AEs related to the target drug, “*c*” represents the number of AE reports for other drugs, and ‘*d*’ represents the number of reports for other AEs related to those other drugs. The ROR value, 95% confidence interval (CI), proportional reporting ratio (PRR), and *χ*^2^ test results were calculated via the corresponding formulas. The calculation formulas are as follows:


ROR=(a/c)/(b/d)

$$\begin{array}{c} 95\%CI\;for\;ROR=e^{\ln\;ROR\pm 1.96\sqrt{1/a+1/b+1/c+1/d}} \end{array}$$

$$\begin{array}{c} \text{P}RR=[a/(a+c)]/[b/(b+d)] \end{array}$$

$$\begin{array}{c} \chi^{\text{2}}=(ad-bc{)}^{\text{2}}(a+b+c+d)/[(a+b)(c+d)(a+c)(b+d)] \end{array}$$

The criteria for detecting vigilance signals are as follows: AE report count ≥ 3; reports with <3 cases are not clinically significant; and the lower limit of the 95% CI for the ROR > 1.^[15]^

## 3. Results

### 3.1. Annual reports of AE in children using growth hormones

**Figure 1.**
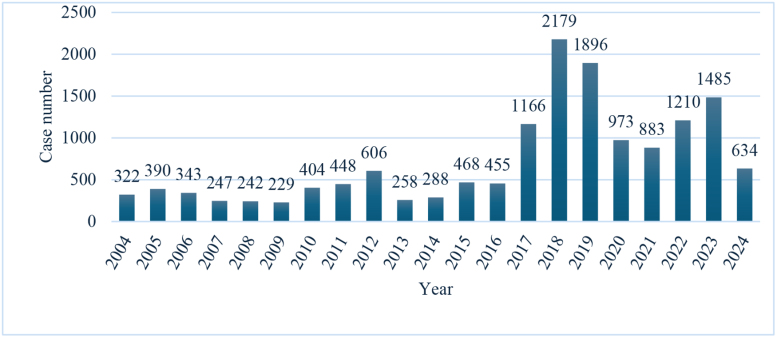
Number of AE reports for children using growth hormone from the first quarter of 2004 to the first quarter of 2024. AE = adverse event.

This study selected AE data related to the use of growth hormones in children under 18 years old from the first quarter of 2004 to the first quarter of 2024. The annual number of reports is shown in Figure 1, which indicates a general increasing trend in the number of reports over the years.

### 3.2. Basic information on AE in children using growth hormones

After searching and filtering, a total of 15,126 AE reports were obtained for children under 18 years of age, with GH as the primary suspected drug. The clinical characteristics of the patients are statistically described in Table 1. In terms of sex distribution, the proportion of females (61.61%) was greater than that of males (36.39%). In terms of age, the age group of 6 to 12 years had the highest number of reports (48.87%). The top 5 reporting countries were the United States, Japan, Colombia, Canada, and Argentina, with the United States having the highest number of reports (62.68%). In terms of clinical outcomes for patients, approximately 20.37% of reports of serious AEs associated with growth hormone were noted, with hospitalization being the most common outcome. The majority of reports were submitted by consumers (50.20%), followed by other healthcare professionals (42.86%). Not specified accounted for 0.47%, and the remaining 6.47% were from other sources.

**Table 1:** Basic information of AEs of growth hormone used by children.

Characteristics	Case number	Case proportion (%)
Gender		
Male	5504	36.39
Female	9319	61.61
Unknown	303	2.00
Age		
≤5	2070	13.69
6–12	7392	48.87
13–18	5664	37.45
Product name (top 5)		
Genotropin	6936	45.85
Omnitrope	1941	12.83
Norditropin	1308	8.65
Nutropin aq	1203	7.95
Saizen	1047	6.92
Indication (top 5)		
Growth hormone deficiency	3861	25.53
Unknown indication	1849	23.32
Short stature	1243	8.22
Hypopituitarism	1181	7.81
Turner’s syndrome	427	2.82
Reported countries (top 5)		
America	9481	62.68
Japan	586	3.87
Colombia	485	3.21
Canada	357	2.36
Argentina	332	2.19
Outcome		
Unknown	7991	52.83
Other outcome	3941	26.05
Hospitalization	2389	15.79
Death	376	2.49
Life-threatening	230	1.52
Disability	86	0.57

AE = adverse event.

### 3.3. Results of signal detection

An analysis was conducted on the 15,126 AE reports with growth hormone as the primary suspected drug, and the ROR method was used to calculate and screen for positive signals. Those signals that were unrelated to the drug, such as product issues, social environment factors, various injuries and poisonings, procedural complications, and various surgeries and medical procedures, were excluded. In the end, 10,559 cumulative AE reports were obtained, resulting in 361 positive signals that spanned 20 different system organ-class (SOCs). The results revealed that the top 5 SOCs on the basis of report numbers were general disorders and administration site conditions (33.4%), investigations (19.97%), musculoskeletal and connective tissue disorders (13.47%), nervous system disorders (13.06%), and metabolism and nutrition disorders (3.55%), as shown in Table 2.

**Table 2:** Number of AE signals by system organ class in children using growth hormone.

SOC (system organ class)	Signal count	Report count	Percentage
General disorders and administration site conditions	41	3527	33.40
Investigations	112	2109	19.97
Musculoskeletal and connective tissue disorders	49	1422	13.47
Nervous system disorders	13	1379	13.06
Metabolism and nutrition disorders	17	375	3.55
Neoplasms benign, malignant and unspecified	28	346	3.28
Infections and infestations	13	306	2.90
Respiratory, thoracic and mediastinal disorders	8	257	2.43
Endocrine disorders	16	187	1.77
Eye disorders	8	140	1.33
Renal and urinary disorders	8	98	0.93
Psychiatric disorders	4	95	0.90
Congenital, familial and genetic disorders	14	90	0.85
Reproductive system and breast disorders	10	67	0.63
Skin and subcutaneous tissue disorders	6	47	0.45
Ear and labyrinth disorders	3	34	0.32
Gastrointestinal disorders	5	32	0.30
Cardiac disorders	3	25	0.24
Immune system disorders	1	13	0.12
Blood and lymphatic system disorders	2	10	0.09

AE = adverse event, SOC = system organ-class.

The effective signals for GH were ranked according to the number of AE reports. Among the most common AEs were injection site pain (1088 cases), headache (987 cases), and arthralgia (334 cases), as indicated in Table 3. Sorted by ROR, the factors with higher relevance include abnormalities in insulin-like growth factor, elevated blood urea nitrogen/creatinine ratio, increased bone density, epiphysiolysis, and neoplasm recurrence, as shown in Table 4. Among the positive signals not mentioned in the product labeling, the top 5 were elevated blood urea nitrogen/creatinine ratio, increased bone density, decreased vitamin D, sleep apnea syndrome, and papilloedema.

**Table 3:** Top 20 AE reports in children using growth hormone.

PT (preferred term)	SOC (system organ class)	Report count	ROR (95% CI)	PRR (*χ*^2^)
Injection site pain	General disorders and administration site conditions	1088	8.89 (8.30–9.53)	8.18 (5541.07)
Headache	Nervous system disorders	987	3.40 (3.18–3.64)	3.20 (1396.24)
Arthralgia	Musculoskeletal and connective tissue disorders	334	3.51 (3.13–3.94)	3.44 (525.68)
Injection site hemorrhage	General disorders and administration site conditions	309	12.79 (11.21–14.61)	12.49 (2355.28)
Injection site bruising	General disorders and administration site conditions	305	18.07 (15.70–20.80)	17.64 (3094.27)
Pain in extremity	Musculoskeletal and connective tissue disorders	253	3.40 (2.98–3.87)	3.35 (377.38)
Pain	General disorders and administration site conditions	241	1.57 (1.38–1.79)	1.56 (46.26)
Malaise	General disorders and administration site conditions	196	1.45 (1.25–1.67)	1.44 (25.23)
Insulin-like growth factor increased	Investigations	192	315.88 (199.23–500.82)	310.86 (5567.41)
Scoliosis	Musculoskeletal and connective tissue disorders	135	16.74 (13.59–20.61)	16.56 (1296.53)
Blood glucose increased	Investigations	134	3.05 (2.55–3.64)	3.02 (164.74)
Growth retardation*	Musculoskeletal and connective tissue disorders	103	4.05 (3.30–4.98)	4.03 (206.24)
Papilloedema	Eye disorders	99	7.49 (6.02–9.33)	7.44 (443.83)
Insulin-like growth factor decreased	Investigations	98	290.85 (155.91–542.57)	288.49 (2804.33)
Sleep apnea syndrome*	Respiratory, thoracic and mediastinal disorders	98	12.95 (10.24–16.37)	12.85 (758.50)
Syncope*	Nervous system disorders	87	1.51 (1.22–1.88)	1.51 (13.91)
Back pain	Musculoskeletal and connective tissue disorders	84	1.61 (1.30–2.01)	1.61 (17.96)
Intracranial pressure increased	Nervous system disorders	81	4.49 (3.56–5.67)	4.47 (188.81)
Blood alkaline phosphatase increased	Investigations	81	4.44 (3.52–5.60)	4.42 (185.56)
Hypoglycaemia	Metabolism and nutrition disorders	77	1.63 (1.29–2.05)	1.59 (16.96)

AE = adverse event, CI = confidence interval, PRR = proportional reporting ratio, PT = preferred terms, ROR = reporting odd ratio, SOC = system organ-class.

* Indicates that the drug instructions did not mention it.

**Table 4:** The top 20 AE positive signal intensities for children using growth hormone.

PT (preferred term)	SOC (system organ class)	Report count	ROR (95% CI)	PRR (*χ*^2^)
Insulin-like growth factor increased	Investigations	192	315.88 (199.23–500.82)	310.86 (5567.41)
Insulin-like growth factor decreased	Investigations	98	290.85 (155.91–542.57)	288.49 (2804.33)
Blood urea nitrogen/creatinine ratio increased*	Investigations	43	155.26 (75.67–318.56)	154.71 (1109.46)
Bone density increased*	Investigations	3	63.78 (39.76–102.33)	63.52 (1038.21)
Epiphysiolysis	Musculoskeletal and connective tissue disorders	61	36.092 (25.06–51.99)	35.91 (964.97)
Neoplasm recurrence	Neoplasms benign, malignant and unspecified	76	34.391 (24.90–47.50)	34.18 (1174.75)
Vitamin D decreased*	Investigations	55	33.13 (22.75–48.25)	32.99 (828.97)
Osteochondrosis	Musculoskeletal and connective tissue disorders	68	28.03 (20.25–38.78)	27.87 (932.32)
Blood creatinine decreased	Investigations	75	24.19 (17.93–32.63)	24.05 (937.46)
Injection site bruising	General disorders and administration site conditions	305	18.07 (15.70–20.80)	17.64 (3094.27)
Scoliosis	Musculoskeletal and connective tissue disorders	135	16.74 (13.59–20.61)	16.56 (1296.53)
Sleep apnea syndrome*	Respiratory, thoracic and mediastinal disorders	98	12.95 (10.24–16.37)	12.85 (758.50)
Blood thyroid stimulating hormone increased	Investigations	48	12.89 (9.22–18.02)	12.85 (366.87)
Injection site pain	General disorders and administration site conditions	1088	8.89 (8.30–9.53)	8.18 (5541.07)
Papilloedema*	Eye disorders	99	7.49 (6.02–9.33)	7.44 (443.83)
Injection site mass	General disorders and administration site conditions	68	5.69 (4.39–7.36)	5.66 (218.28)
Type 2 diabetes mellitus	Metabolism and nutrition disorders	48	4.89 (3.61–6.62)	4.87 (125.08)
Intracranial pressure increased	Nervous system disorders	81	4.49 (3.56–5.67)	4.47 (188.81)
Blood alkaline phosphatase increased	Investigations	81	4.44 (3.52–5.60)	4.42 (185.56)
Idiopathic intracranial hypertension	Nervous system disorders	55	4.41 (3.33–5.85)	4.40 (124.19)

AE = adverse event, CI = confidence interval, PRR = proportional reporting ratio, PT = preferred terms, ROR = reporting odd ratio, SOC = system organ-class.

* Indicates that the drug instructions did not mention it.

## 4. Discussion

Growth hormone (GH) medications are widely used in children to treat various causes of short stature.^[16]^ While rhGH is clinically effective, its safety concerns and long-term effects on children have garnered significant attention. This study analyzed the characteristics of AEs associated with growth hormone medications in children via the FAERS database, employing the ROR and PRR methods.

### 4.1. Analysis of adverse events related to growth hormone medications

From the first quarter of 2004 to the first quarter of 2024, the number of AEs reported for growth hormone use in children under 18 years of age generally increased, with a marked rise starting in 2017 and peaking in 2018, followed by a slight decline. The primary reasons for this trend include the prolonged time since the drug’s market entry, the increasing variety of medications, and the growing clinical application of growth hormone, leading to an increase in AE reports. However, public awareness of adverse reactions to growth hormones has also increased, resulting in more cautious prescribing practices among clinicians, better patient monitoring, and education, ultimately stabilizing the number of AE reports. Among the 15,126 AE reports included in this study, most were from children aged 6 to 12 years, the primary population treated for short stature. The sex distribution revealed a significantly greater number of females than males. There have been no reports indicating whether there is a difference in sensitivity to growth hormone medications between sexes, which warrants further exploration. Approximately 20.37% of reported AEs were classified as serious, indicating the presence of a certain risk.

### 4.2. Analysis of common AE signals related to growth hormone-mediated effects

The study identified 10,559 AE reports and 361 signals, covering 20 SOCs. The top 5 SOCs on the basis of report numbers were general disorders and administration site conditions, investigations, musculoskeletal and connective tissue disorders, nervous system disorders, and metabolism and nutrition disorders. The most commonly reported AEs include various reactions at injection sites, headaches, and abnormal laboratory findings. Additionally, risks such as increased intracranial pressure, slipped capital femoral epiphysis, and scoliosis are noted, which is consistent with common adverse reactions listed in the prescribing information and current research.^[17]^

Tumor-related adverse reactions are a primary concern for parents, and this study revealed that malignant and unspecified tumor reactions accounted for 3.28% of the reports. Growth hormone is known to promote anabolic metabolism and cell mitosis, with 1-insulin-like growth factor-1 exhibiting antiapoptotic properties, theoretically increasing the risk of tumorigenesis.^[18]^ This does not mean that GH directly leads to tumorigenesis, because FAERS data cannot provide evidence of causality. The National Cooperative Growth Study and the Pfizer International Growth Database (KIGS), along with the findings of most studies, suggest that growth hormone does not increase the risk of malignant tumors.^[19-22]^ A recent article in The Lancet indicated that rhGH treatment does not affect overall cancer risk, although these studies have limitations.^[23]^ The relatively high reporting rate of tumor-related adverse effects may be because individuals tend to be more sensitive to tumor-related health concerns than to other AEs, and patients in tumor-susceptible populations may already have existing tumors, potentially leading to secondary malignancies. The causal relationship between growth hormone and adverse tumor effects requires further research.

### 4.3. Analysis of newly suspected AE signals related to growth hormone medications

Among the top 20 reporting numbers and positive signal strengths based on preferred terms, 8 new suspected positive signals were identified that were not documented in the product labeling. These included growth retardation, sleep apnea syndrome, syncope, increased bone density, papilloedema, and some abnormal laboratory results. Research indicates that short-term risks for sleep apnea syndrome often occur in children with baseline obstructive symptoms or after upper respiratory infections. In contrast, growth hormone treatment does not appear to impact sleep-disordered breathing.^[24]^ Both syncope and papilloedema may be part of a series of complications caused by intracranial hypertension. Growth retardation could reflect individual differences in response to growth hormone therapy, with some children showing no significant effects or other underlying conditions causing stunted growth. Abnormal laboratory results may be related to the influence of growth hormone on endocrine function and metabolic growth, necessitating ongoing surveillance for new AEs associated with growth hormone medications, particularly those that are novel and subtle.

### 4.4. Study limitations

This study relied on the FAERS database to conduct signal detection for AEs related to growth hormone medications in children. As a voluntary reporting system, it may contain duplicate reports, incomplete data, weak causal relationships, or even erroneous information. The reporting countries were predominantly the United States, Japan, and Colombia, with lower representation from other countries, introducing a potential racial bias and underscoring the need for more safety monitoring data across diverse populations. While the study indicated an incidence of benign, malignant, and unspecified tumors, this finding contradicts some existing research, suggesting the need for in-depth and long-term follow-up studies to determine any causal relationship between growth hormone and tumors. Additionally, FAERS data have inherent limitations including spontaneous reporting bias, as reports originate from multiple countries with different reporting practices. Uncontrollable confounders such as drug dose, treatment duration, comorbidities, and concomitant medications cannot be adequately adjusted. Moreover, the lack of denominator data (total number of patients exposed to growth hormones) prevents calculation of true incidence rates of AEs.

## 5. Conclusion

This study is based on the FAERS database and utilizes the ROR method to mine and analyze the AE signals of GH. The results show that the safety profile of GH in the real world is generally consistent with the findings from clinical trials, and the overall safety is good. However, many issues persist, particularly regarding its long-term effects on children, thus highlighting the importance of rigorous monitoring and follow-up. This study supplements the safety information of GH in the real world and provides important references for the safe use of these drugs in clinical practice. These findings can help doctors better monitor and manage adverse drug reactions in patients during clinical practice, thereby optimizing treatment plans.

## Author contributions

**Conceptualization:** Yao Fei.

**Data curation:** Jianan Bao.

**Funding acquisition:** Jianan Bao.

**Investigation:** Wan Xu.

**Methodology:** Wan Xu.

**Writing – original draft:** Yao Fei.

**Writing – review & editing:** Wan Xu.
